# Effects of Chronic Obstructive Pulmonary Disease and Obstructive Sleep Apnea on Cognitive Functions: Evidence for a Common Nature

**DOI:** 10.1155/2014/768210

**Published:** 2014-02-06

**Authors:** Georgia Andreou, Filippos Vlachos, Konstantinos Makanikas

**Affiliations:** Department of Special Education, University of Thessaly, Argonafton & Filellinon, 38221 Volos, Greece

## Abstract

Patients with chronic obstructive pulmonary disease (COPD) and obstructive sleep apnea syndrome (OSAS) show similar neurocognitive impairments. Effects are more apparent in severe cases, whereas in moderate and mild cases the effects are equivocal. The exact mechanism that causes cognitive dysfunctions in both diseases is still unknown and only suggestions have been made for each disease separately. The primary objective of this review is to present COPD and OSAS impact on cognitive functions. Secondly, it aims to examine the potential mechanisms by which COPD and OSAS can be linked and provide evidence for a common nature that affects cognitive functions in both diseases. Patients with COPD and OSAS compared to normal distribution show significant deficits in the cognitive abilities of attention, psychomotor speed, memory and learning, visuospatial and constructional abilities, executive skills, and language. The severity of these deficits in OSAS seems to correlate with the physiological events such as sleep defragmentation, apnea/hypopnea index, and hypoxemia, whereas cognitive impairments in COPD are associated with hypoventilation, hypoxemia, and hypercapnia. These factors as well as vascocerebral diseases and changes in systemic hemodynamic seem to act in an intermingling and synergistic way on the cause of cognitive dysfunctions in both diseases. However, low blood oxygen pressure seems to be the dominant factor that contributes to the presence of cognitive deficits in both COPD and OSAS.

## 1. Introduction

Chronic obstructive pulmonary disease (COPD) and obstructive sleep apnea syndrome (OSAS) represent two of the most common chronic respiratory disorders. COPD is characterized by airflow limitation that is not fully reversible, while OSAS by periodic complete or partial upper airway obstruction during sleep [1, 2]. Studies have shown that the occurrence of both diseases among adults is advancing along with aging. As the global population ages, the prevalence of COPD and OSAS will increase in future and as a result more and more patients will be in need of health care [1, 3].

Furthermore, it has been suggested that COPD and OSAS affect cognitive functions [4, 5]. However, there are different opinions on which cognitive abilities are affected by these two respiratory disorders and on the exact cause of cognitive deficits. Comparing studies is difficult because of design study variability, differences in severity of disease, mode of selection of patients and control groups, sample size, and respective study inclusion and exclusion criteria and because of different criteria that are used to assess the severity of syndromes [6, 7]. Other variables such as the neuropsychological battery chosen, the treatment prescribed (e.g. oxygen supply), corticoid prescriptions [6], and different group ages are also confounding factors [8]. Nevertheless, the present study is an attempt to compare the performance of COPD and OSAS patients in common neuropsychological tests according to severity so as to understand better the role of respiratory/lung disturbances, blood gas levels, and sleep defragmentation on neurocognitive functions.

## 2. Chronic Obstructive Pulmonary Disease (COPD)

Chronic obstructive pulmonary disease (COPD) is a progressive disease characterized by the presence of airflow obstruction secondary to emphysema or chronic bronchitis [9]. COPD does not cause a fully reversible airway limitation due to chronic inflammatory process in the pulmonary tissue that often results in breathlessness for the patients, cough [10, 11], and excessive mucus production [12, 13]. In the United States, COPD is the fourth leading cause of death [13], after heart disease, cancer, and stroke [14], whereas its prevalence increases with aging [15, 16]. The occurrence of COPD in general population is between 2.83% [15] and 6.9% [17], while in ages older than 35 years old this percentage amounts to 17.4% in developed countries [16]. More COPD patients are between their fifth and sixth decade of life [10] and there is a higher incidence of the disease among males than females [16, 18].

Tobacco smoking is the major cause of the disease, although only a minority of smokers develop clinically significant symptoms [10]. Other factors, such as indoor and outdoor air pollution, infection in childhood, asthma, genetic factors [1, 9] and occupational dust have been reported to contribute to the development of COPD [10]. COPD is associated with an increased mortality and morbidity implications such as lung cancer, anemia [10], pulmonary hypertension, polycythaemia, peripheral oedema, cardiovascular complications, obstructive sleep apnea, chronic infections, and musculoskeletal disorders (e.g., osteopenia and muscle atrophy) [1, 19] as well as nutritional depletion that is caused by increased metabolism during breathiness episodes [11, 13]. Patients with COPD are significantly more likely to report symptoms such as insomnia and difficulty in initiating and maintaining sleep [20]. Moreover, they have a higher rate of depression and anxiety compared to general population [21]. COPD has also been associated with reduced patients' quality of life due to restriction of activities and limitation of social life [22].

The treatment of COPD patients is based on a combination of bronchodilators, corticosteroids, antibiotics, and mucolytics which have been found to improve lung functions, COPD symptoms and morbidity and mortality caused by extrapulmonary effects. Drug use has also been found to reduce exacerbation rates (major changes in patient's symptoms or their requirement for drug therapy) and the length of hospital stay [11, 12]. Furthermore, it has been found that drugs improve patients' quality of life [11] and mood [23]. However, the most effective treatment is smoking cessation which delays disease progression [11] or returns lung functioning to normal, if COPD is diagnosed at early stages [12]. Some other kinds of treatment such as pulmonary rehabilitation, lung volume reduction surgery (LVRS) [10], long-term oxygen therapy (LTOT) [12], and continuous positive airway pressure (CPAP) [14] are also effective in reducing patients' morbidity and mortality.

### 2.1. Definition

COPD is determined on the basis of chronic respiratory symptoms, including irreversible airflow limitation associated with dyspnea and particularly with exertion, productive cough, and wheeze [11, 14]. The pathological processes in COPD include inflammatory damage of the large airways (trachea, bronchi) and small airways (bronchioles) and often alveolar dysfunction of small airways [12, 13]. The glandular hypertrophy and the reduced number of cilia that are observed in large airways cause increased cough [12]. However, the majority of symptoms in COPD patients is based on the reduced ability of lungs to empty sufficiently. Inspiratory muscle weakness, lung hyperinflation, and increased ventilatory demand relative to capacity contribute to the presence of dyspnea [12, 24]. On the other hand, alveolar dysfunction causes impairment in gas exchange (hypoventilation) and ventilation-perfusion mismatching, which result in hypoxemia and hypercapnia [12, 25].

The diagnosis of COPD is based on a typical history of persistent and progressive symptoms on risk factors for COPD and on assessment of physiologic measures of lung function [12]. The most commonly used test for the evaluation of lung dysfunction is spirometry [10]. The severity of the disease is based on spirometric criteria measuring the forced expiratory volume in 1 sec (FEV_1_) and the ratio of FEV_1_ to forced vital capacity (FVC) after bronchodilator administration [1, 11]. The stage of severity of COPD is determined according to 2010 Global initiative for Obstructive Lung Disease (GOLD) guidelines. Therefore, COPD is categorized as mild when FEV_1_ is ≥80%, moderate when FEV_1_ is among ≤50–<80%, severe when FEV is ≤30%–50%, and very severe when COPD patients are presented with an FEV_1_ <30%. Diagnosis also demands FEV_1_/FVC to be <0.70 [1, 26]. A useful feature for confirmation of the diagnosis is that COPD patients' lung functions do not return to normal after bronchodilator administration, in contrast to patients with other breathing disorders (e.g., asthma) [12].

Severity of hypoxia in COPD is defined by blood oxygen levels. A mild stage of severity is considered the one with an oxygen partial pressure (PaO_2_) between 50 mmHg and 80 mmHg [27]. At this level, there is complete compensation and general function is barely altered. Oxygen partial pressure between 35 and 50 mmHg is generally considered moderate hypoxia, a state which leads to a negative impact on cognition. Finally, when PaO_2_ is below 35 mmHg the hypoxia is considered severe [6]. As regarding hypercapnea, it is characterized as abnormal when carbon levels exceed the normal range of 35 mmHg to 45 mmHg [27].

### 2.2. Effects of COPD on Cognitive Functions

Chronic obstructive pulmonary disease has been found to cause a general cognitive decline [28, 29] especially in cognitive functions such as attention, psychomotor speed, memory and learning, visuospatial and constructional abilities, executive functions, and language skills [28, 30]. A summary of relevant studies and their results is presented in Table 1.

#### 2.2.1. Attention

Attention in COPD patients is one of the most widely studied cognitive functions. Impairments on vigilance and sustained visual attention have been documented by several studies [31–33]. More specifically, a study showed that severe-to-very severe COPD patients with mild hypoxia performed poorly on visual sustained and selective attention tasks [34]. Other studies have proved that severe COPD patients with mild-to-moderate hypoxia performed poorer than healthy controls on alerting, orienting tasks, and sustained attention tasks [35–37]. Attention decline has also been observed in severe COPD patients with moderate hypoxia and hypercapnia [38].

Moreover, a research on moderate and severe COPD patients with mild severity oxygen pressure and moderate severity nocturnal oxygen desaturation found impairments in selective and sustained attention [39]. Poor performance in attention tasks has been demonstrated even in nonhypoxemic severe COPD patients [40, 41].

On the other hand, there are studies on COPD patients which found a mild decline in attention abilities [42] and an intact visual immediate attention even in severe COPD patients [43]. A more recent study on severe COPD patients who were mildly hypoxemic noted that the prevalence of visual and verbal attention decline was 14.9% and 2.2%, respectively [44]. Overall, the aforementioned studies have demonstrated that COPD patients exhibit poorer performance than healthy controls in several domains of attention.

#### 2.2.2. Psychomotor Speed

A decline in psychomotor speed was evidenced by a variety of research studies. It has been shown that 26% of a group of very severe COPD patients with mild hypoxia were impaired in psychomotor speed [9]. Another study on severe COPD patients with mild-to-moderate hypoxia proved that they achieved statistically significant low scores in time reaction tests [35]. Furthermore, it has been shown [45] that severe COPD patients who were oxygen and non-oxygen dependent exhibited poor performance in psychomotor speed tests. This finding was consistent with the findings of a study [46] in which nonhypoxemic severe COPD patients had low performance in a psychomotor speed test. On the other hand, other researchers found [47] that moderate COPD patients, with mild hypoxia, performed within average range in a test which assesses psychomotor speed. Finally, there is a study [41] which failed to document differences between healthy controls and hypoxemic or nonhypoxemic patients in psychomotor speed tests. Up to now research has yielded contradicting data on the association of COPD and psychomotor skills. Therefore, more research is needed in order to shed light on the impact of COPD on psychomotor skills.

#### 2.2.3. Memory and Learning

Memory dysfunctions are common in COPD patients. The majority of researchers have documented deficits in verbal short-term and long-term memory [32, 41], in visual memory [33], and in spatial memory [31].

Severe-to-very severe COPD patients have usually been presented with a significant short-term verbal memory decline. COPD patients' performance in visual span tests was found impaired in contrast to their performance in immediate visual memory tests which was found intact [48]. There is also a study [38] which has documented a verbal memory decline in a patient with severe COPD and moderate hypoxia, who was in need for lung volume reduction surgery. In patients with severe COPD and mild-to-moderate hypoxemia statistically significant differences in verbal and visual learning have been found when compared to healthy controls [35]. A research in severe COPD patients has documented impairments in delayed verbal memory [37]. In addition, poor performance in verbal memory and delayed recall was observed in nonoxygen and oxygen dependent patients with severe COPD [41, 45], although a decline in immediate visual memory was rarely found [41, 44].

Some other studies have reported memory impairments in a significant percentage of COPD patients of their samples. It was found [49] that 80% of patients with severe COPD and mild-to-moderate hypoxia performed poorer than healthy controls in verbal short-term and long-term memory tests, while 38.1% of them presented a specific verbal memory profile. Another study [9] found that 50% of very severe patients with mild-to-moderate hypoxia had impaired immediate memory and 44% of them had difficulties in their long-term retrieval ability. A specific pattern of cognitive deterioration was found in 48% of patients with COPD, which included a dramatic verbal memory decline [43]. A recent study on severe COPD patients with mild hypoxia found that 37.3% of them was impaired in terms of short-term memory and 26.1% obtained low scores in long-term memory tests [44]. In another study [50], it was noted that 30% of COPD patients had impaired memory which was confined to immediate verbal memory.

Finally, there is a number of studies that failed to document any memory deficits in COPD patients. Moderate COPD patients' performance with mild hypoxia was found within average normal range in memory tests [47], while another study [39] on moderate COPD patients with mild oxygen blood pressure did not demonstrate notably poor scores in verbal learning memory tests. Moreover, short-term and long-term verbal memories have been found within normal range in nonhypoxemic severe COPD patients [46, 48]. Based on the discrepancy among the results of the aforementioned studies the issue of learning and memory abilities in COPD patients should be further examined in combination with the severity of hypoxia that these patients display because it seems that hypoxia has a negative impact on memory and learning.

#### 2.2.4. Visuospatial and Motor Constructional Abilities

A decline in constructional abilities is very common among COPD patients [32]. Severe COPD patients with mild hypoxemia have been presented with impaired constructional abilities [37, 51]. Another study has noted a decline in visuoconstructional abilities in 40.3% of severe COPD patients with mild hypoxia [44]. On the other hand, there are researches in which a normal performance was found in hypoxemic and nonhypoxemic COPD patients [41] as well as in severe-to-very severe COPD patients with mild hypoxia [48]. Despite the slight discrepancy among the findings of studies on the impact of COPD on visuospatial and motor constructional abilities, most of them provide evidence for impaired visuoconstructional abilities in COPD patients.

#### 2.2.5. Executive Functions

The presence of executive dysfunctions among COPD patients is still equivocal. There have been studies that found impairments in problem solving ability, abstraction abilities, and deductive thinking [40]. More specifically, studies on severe-to-very severe COPD patients with mild hypoxia have demonstrated mild impairments in executive functions [37]. A research on severe COPD patients with mild-to-moderate hypoxia showed impairments in logical thinking but not in general executive functioning [35]. A study [52] on severe COPD patients with mild hypoxemia also found a mild decline in letter fluency; however, this was not at a clinically impaired range. Concerning nonhypoxemic severe COPD patients, their performance in tests that assess mental flexibility was found poor [46]. Another study [47] found that moderate COPD patients' performance with mild hypoxia in tests that require mental shift ability was below average. It was also found [9] that 31% of very severe COPD patients with mild-to-moderate hypoxia exhibited impaired performance in tests measuring executive functioning. In addition, in a group of mildly hypoxemic patients with severe COPD, it was noted that 40.3% of them performed poorly in copying complex drawings and 11.2% in Raven progressive matrices, a test examining nonverbal reasoning.

Nonetheless, there has been research, though limited, that either failed to find any significant poor performance in executive functioning in severe COPD patients with mild hypoxia [48] or noted that COPD patients' performance was similar to controls in executive functioning tests [41]. It must be stressed, however, that most studies support the notion that COPD is usually associated with executive dysfunctions.

#### 2.2.6. Language Abilities

There has been a significant number of studies that have documented serious language impairments assessed with verbal fluency tests in COPD patients [33, 36]. A recent study found a language decline in nonhypoxemic and hypoxemic COPD patients in selected tests of verbal attainment [40]. Another study found that 48% of severe COPD patients presented a specific pattern of cognitive deterioration which included a dramatic decline in verbal fluency [43]. On the other hand, there is a research which has shown that only 10.4% of severe COPD patients with mild hypoxia showed sentence construction decline and 7.5% a verbal fluency impairment [44].

Nonetheless, the presence of language dysfunctions in COPD patients is still controversial. There is a study which found that severe COPD patients with mild hypoxia performed low in verbal fluency tests, although this was not at a clinically impaired range [52]. Similarly, it was demonstrated [47] that moderate COPD patients with mild hypoxia performed within average range in the aphasia screening test, while in a verbal fluency test their performance was towards the low end of the average range.

Moreover, a study [9] on very severe COPD patients with mild-to-moderate hypoxia found normal scores in vocabulary tests. It has also been shown that severe COPD patients' performance in crystallized intelligence (knowledge and vocabulary) [35] and in language (verbal fluency, sentence construction) was within normal range [48]. Finally, there is a research [41] which reported that hypoxemic and nonhypoxemic COPD patients' performance was similar to controls in verbal production and verbal competence tests. From the aforementioned studies (for details see Table 1) it is apparent that the unclear association of COPD and language abilities can be partially attributed to differences in the severity of disease, mode of selection of patients and control groups, and on different criteria that are used to assess the severity of syndromes.

## 3. Obstructive Sleep Apnea-Hypopnea Syndrome

Obstructive sleep apnea syndrome (OSAS) is part of a spectrum of sleep related breathing disorders which also includes central sleep apnea (CSA) (apnea without respiratory effort), mixed sleep apnea (episodes of obstructive sleep apnea and central sleep apnea), upper airway resistance syndrome (increased respiratory effort without apnea or hypopnea), and snoring [3, 53]. It affects 2% of women and 4% of men in middle age adults [54]. A recent study on a population-based sample of subjects aged 30 to 70 years found that 19% of men and 15% of women present hypopnea and apnea episodes above normative index [55].

OSAS is characterized by a perturbation of the pharyngeal dilator muscles. More specifically, it occurs when the muscles relax during sleep, causing soft tissue in the back of the throat to collapse and block the upper airway and it is associated with or without faulty upper airway anatomy such as macroglossia, hypertrophy of tonsils, and long uvula [2, 3]. Other risk factors that enhance the probability of OSAS are obesity, increased neck circumference, positive family history, male postmenopausal status, Down syndrome, Pierre-Robin syndrome, alcohol consumption before bedtime, tobacco and hypnotics use, and sleeping in supine position [2, 56]. These factors result in episodes of complete or partial upper airway obstruction during sleep and in increased sleep arousals that terminate the apneic episodes. These disturbing events while sleeping cause decreased oxygen saturation as well as sleep fragmentation [3, 57]. It has been found that stage 2 of sleep increases, while stage 1, stage 3, stage 4, and REM sleep decrease in OSAS [3, 56], although there are different opinions on the decreased total time of REM sleep [58, 59]. This abnormal sleep architecture makes sleep lighter and less restorative [56].

The most well-known symptom of obstructive sleep apnea/hypopnea syndrome is excessive daytime sleepiness [60], although some studies failed to report daytime somnolence [61]. Daytime sleepiness is usually assessed with multiple sleep latency test (MSLT), maintenance of wakefulness test (MWT), and with a series of questions included in the Epworth sleepiness scale (ESS) which evaluate patients' condition in common everyday situations [62]. The majority of patients with OSAS report that they fall asleep in quiet and monotonous situations such as watching television, reading a book, or driving a vehicle [56]. Fatigue, nonrefreshing sleep, insomnia, loud snoring, gasping, choking, and reports of breathing interruptions by bed partner are common daytime and nocturnal symptoms of sleep apnea [63]. Poor quality of life, poor interpersonal relationships, and low work and school efficiency have been observed in OSAS patients [5]. Moreover, patients with OSAS have a high frequency of psychopathology such as depression and hypochondriasis [64]. In addition, patients with OSAS present increased morbidity due to high rates of cardiovascular diseases and hypertension [65] as well as increased mortality [5].

The treatment of OSAS is based on restoring the upper airway flow. For that reason, the most effective treatment for patients with craniofacial abnormalities is surgical treatment. In the rest of OSAS patients the most effective treatment is the continuous positive pressure therapy (CPAP) which consists of an air pressure generating device and a fitting mask that is applied over the nose or the mouth of the patient. The positive air pressure maintains upper airway patency and prevents upper airway obstruction, while the patients are asleep [2, 3]. CPAP therapy has been shown to improve nocturnal breathing, oxygen saturation [3], and mean blood pressure [66]. As a result, it decreases hypertension and the risk of cardiovascular and cerebrovascular events [65]. CPAP therapy in most cases decreases daytime sleepiness [64] and improves OSAS patients' mood [67] and quality of life [68]. However, these positive effects have not always been reported [61].

Recent studies have shown that CPAP treatment is also related to cognitive improvements [69]. In a clinical review [56], it has been concluded that CPAP is effective in reducing symptoms of sleepiness and improves some cognitive functions.

### 3.1. Definition

Obstructive sleep apnea syndrome (OSAS) is defined as an obstruction of airflow for 10 seconds or longer, while both apneas and hypopneas are observed in the syndrome. Apnea is characterized by the cessation of airflow (decrements in airflow of ≥90%) for 10 seconds or more [2, 3, 53]. On the other hand, hypopnea is usually characterized by a reduction of ≥30% in airflow for 10 seconds associated with a ≥3% decrease in oxygen saturation or arousal [70]. According to the American Academy of Sleep Medicine, hypopnea is defined as a decrease of ≥30% in airflow, for a period lasting at least 10 seconds, followed by ≥4% oxygen desaturation, while at least 90% of the event's duration must meet the amplitude reduction of criteria for hypopnea [2, 71]. The diagnosis of the syndrome is based on daytime and nocturnal symptoms and especially on a full-night polysomnogram [72], which includes electroencephalographic, electro-occulographic electromyographic, oxygen saturation, oral and nasal airflow, respiratory effort, and electrocardiographic and leg movement recordings [2]. OSAS severity is defined according to apnea-hypopnea index (AHI), by calculating the sum of apneas plus hypopneas per hour of sleep. According to apnea/hypopnea index (AHI), OSAS patients are divided into 3 groups of severity: mild OSAS (AHI ≥ 5), moderate OSAS (AHI = 15–30), and severe OSAS (AHI ≥ 30) [2].

### 3.2. Neurocognitive Deficits in Obstructive Sleep Apnea/Hypopnea Syndrome

It has been repeatedly documented that OSAS has a negative impact on a wide range of cognitive functions. Attention, memory, psychomotor speed, visuospatial abilities, constructional abilities, executive functions and language abilities are among the most impaired cognitive domains in OSAS patients [5, 73]. A summary of relevant studies is presented in Table 2.

#### 3.2.1. Attention

Patients with OSAS have been found with impaired attention abilities [68] and more specifically with a decline in vigilance [74, 75] and in complex attention [76]. Research has shown that severe OSAS patients are characterized by diffused impairments in vigilance [53, 60, 64], in selective and sustained attention as well as alertness [53, 77]. In line with the above studies, another study [8] showed that a group of patients with severe OSAS, when compared to controls, had poor performance in tests measuring sustained attention and vigilance. Moderate and severe OSAS patients have been found to show diffused impairments in vigilance [67], as well as in selective, sustained, and divided attention [78].

On the other hand, there have been studies on moderate and severe OSAS patients that failed to find deficits in attention [58, 79]. Moderate OSAS patients (AHI = 20 ± 6) performed at a normal range in neuropsychological tests that assess sustained, divided attention and vigilance [80].

However, a study on mild-to-severe OSAS patients did not document a significant attention deficit on their part [81]. Additionally, other studies [7, 68] found no significant impairments in attention in mild and moderate OSAS patients, compared to healthy participants. From the findings of the aforementioned studies it is apparent that the association between OSAS and attention abilities is unclear, probably related to methodological differences across studies.

#### 3.2.2. Psychomotor Speed

There are a significant number of studies [75, 76, 82] which have reported deficits in psychomotor efficiency among OSAS patients. A study [53] which compared normative data to severe OSAS patients' performance in psychomotor tasks found a decline in psychomotor speed. There has also been evidence for psychomotor speed deficit in moderate and severe OSAS patients [58, 83].

However, differences among severe OSAS patients' and healthy controls' performance in psychomotor speed tests have not always been detected [8]. In a recent study [81], psychomotor deficits in mild-to-severe OSAS patients were not reported. In the same line, a study on patients with mild and moderate OSAS compared to controls did not show any significant poor performance in psychomotor speed tests [7]. The above mentioned studies provide inconclusive results concerning the relationship of OSAS with psychomotor speed which probably has to do with the difference in the amount of severity of the disease in the existing research studies.

#### 3.2.3. Memory and Learning

OSAS patients have repeatedly been presented with serious impairments in memory and learning abilities [82], such as episodic memory [84], short-term memory [69], long-term verbal memory [84], and verbal and visual learning abilities [86]. It has been found that severe OSAS patients' performance was poor in short-term verbal memory tests [53, 87], long-term learning, and memory tests [53] as well as working [88], visual and spatial memory tests [60, 87].

However, not all studies on severe OSAS patients have found memory and learning impairments. In contrast to the above studies that found a serious memory decline in severe OSAS patients, there is a study [8] which showed that severe OSAS patients preserved a good short-term memory, long-term episodic memory, and procedural and working memory and only their performance in immediate recall was found decreased. In line with the above findings, another research study [60] reported no memory decline in severe OSAS patients.

Moderate and severe OSAS patients have been presented with poor verbal immediate, delayed recall and working memory functions [83, 89]. Low scores have been reported on short-term memory and on spatial short-term memory tests in moderate and severe OSAS patients, in contrast to long-term verbal memory ability which was found intact [79].

However, no significant deficits in episodic memory and working memory have been observed in patients with moderate and severe OSAS [58]. Another study found no differences between normal and mild-to-severe OSAS patients in visual memory and verbal working memory [81], while immediate and delayed recall of logical memory as well as spatial working memory were found impaired. In addition, patients with moderate OSAS did not show any decline in visual or verbal memory skills [80]. In sum, data with regard to memory functioning in OSAS patients are mixed with future research still remaining to elucidate the relationship between OSAS and memory and learning abilities.

#### 3.2.4. Visuospatial and Motor Constructional Abilities

A small number of studies have reported poor visuospatial and motor constructional abilities in OSAS patients since research in the field was conducted only in the last decade. Patients with severe OSAS have been found to display visuospatial and constructional impairments [60, 85] and moderate and severe OSAS patients in another study have been found to obtain low scores in tests measuring visuospatial and motor constructional skills [89]. This small body of the existing research provides evidence that OSAS has a negative impact on visuospatial and motor constructional abilities; however, the restricted number of studies done in the field delineates the need for further research in this area.

#### 3.2.5. Executive Functions

Executive abilities including mental flexibility, planning, working memory, analysis, synthesis, and organizational skills have been found seriously impaired in OSAS patients [63, 76, 90]. Executive dysfunctions including low performance in phonemic verbal fluency and letter-number sequencing tasks have been observed in patients with severe OSAS [53, 60, 85, 91]. However, there is a study [8] which failed to find a noteworthy decline in executive functions in severe OSAS patients. Regarding moderate and severe OSAS patients, it has been found that they performed poorly in tests that assess executive functions [67, 83]. On the other hand, there are some studies on moderate and severe OSAS patients that did not report any impairment in executive functions [58, 79]. Mild-to-severe OSAS patients have been documented to have significant impairments in mental shift and planning and minimal decline in phonemic fluency [81].

On the other hand, it has been found that moderate OSAS patients either perform within normal range in executive functioning tests such as the verbal fluency test [68, 80] or that their executive functioning is impaired at a limited amount [7]. It is remarkable that some researchers [92] have found that OSAS patients' performance in some tests measuring executive functions such as the letter verbal fluency [69, 89] were within normal range although their performance was poor in the rest of neuropsychological tests that are sensitive to the detection of executive dysfunctions. One possible explanation is that there is a pattern of both intact and impaired cognitive functions in OSAS patients that make executive impairments difficult to detect [93]. More specifically, it was suggested [88] that there is a specific working memory deficit associated with complex memory tasks and high level memory scanning. Additionally, it has been suggested that deficits in tasks that require high executive functioning are apparent only over the course of the day due to circadian variations or duration of time spent awake [93]. In sum, this review of the literature indicates that the empirical data which examine the relation between OSAS and executive functions are not enough to explain the pattern that executive functions follow in OSAS. Therefore, more research is needed in order to elucidate which are the exact executive functions that are impaired in OSAS and provide an explanation for that.

#### 3.2.6. Language Abilities

Although the bulk of the research conducted on the impact of OSAS on neurocognitive functions focuses on cognitive functions such as attention, memory, and executive functioning rather than on language abilities, there have been some studies on OSAS patients that have shown significant semantic language deficits on their part [73, 87], although this has not always been the case [94]. The fact that more research has been carried out on cognitive abilities other than language is probably associated with the fact that some of them, for example, attention and memory problems, are more easily noticed by patients themselves as well as by their bed partners and doctors than language deficits are. Research on severe OSAS patients' language abilities [53, 85, 91] has shown that they suffer serious problems in verbal tasks and especially in the semantic domain of language. On the other hand, there is a study [60] which failed to find any significant decline in language abilities concerning the semantic domain, while the same study found a serious impairment in the phonemic domain of language of patients with severe OSAS.

Research on moderate and severe OSAS patients has shown that their performance in semantic tasks was similar to healthy controls [89]. Minimal differences between mild-to-severe OSAS patients and healthy volunteers in semantic and phonemic tasks were also documented [81]. Another study found that moderate OSAS patients' language abilities were within normal range [80]. An interesting finding is that mild and moderate OSAS adolescents have shown significantly lower scores in semantic and phonemic language tasks [73].

In sum, the existing research data on language abilities in OSAS patients do not clearly indicate which are the language domains mostly affected by OSAS and to what extent. Therefore, more research is needed in the field taking into account factors other than the severity of symptoms such as the age of the participants because it has been suggested that OSAS occurrence during critical ages of brain growth and development such as childhood and adolescence may cause notable language decline [73].

## 4. Mechanism of Cognitive Impairment in COPD and OSAS Patients

### 4.1. Mechanism of Cognitive Deficits in COPD Patients

It has been suggested that cognitive impairments in COPD patients are caused by independent factors, such as COPD severity parameters (FEV_1_, FEV_1_/FVC) [95], oxygen desaturation (SaO_2_) [96], oxygen partial pressure (PaO_2_), and hypercapnia (PaCO_2_) [97]. More specifically, lung volume impairments defined by FEV_1_/FVC (%), FEV_1_ (%), and FVC (%) have been presented to correlate with performance in tests, such as the Mini-Mental State Test, which examines memory, as well as with orientation, and language skills [27]. It has also been found [98] that low FEV_1_ during middle age was a significant predictor of cognitive disabilities in later life. Moreover, research has shown that more severe COPD patients presented lower scores in cognitive tests than moderate or healthy adults [29]. In other words, the degree of pulmonary decline plays a crucial role on the level of cognitive impairment [99]. However, the majority of researches failed to demonstrate a significant association between lung parameters (FEV_1_, FVC) and cognitive impairments [39, 46, 96].

Another factor that has been found to be related to impaired cognitive functions is low oxygen desaturation (SaO_2_) [22, 100]. It has been shown that there is a strong correlation between low baseline oxygen saturation (≤80%) and cognitive impairment [96]. It has also been found that the risk of cognitive impairment increases with decreasing oxygen desaturation [96]. It has been suggested that low nocturnal desaturation or nocturnal blood gas may explain better the cognitive deficits in a similar way in OSAS patients [46], since hypoxemic COPD patients become more hypoxemic during sleep [25].

The majority of researches documented a correlation between cognitive functions and the degree of hypoxemia defined by arterial oxygen pressure (PaO_2_). More specifically, it has been found that partial pressure of arterial oxygen (PaO_2_) is related to complex attention, psychomotor speed, executive functioning, constructional abilities [44, 47], and visual and verbal short-term memory [47]. The crucial impact of blood oxygen level on cognitive functions can also be proved by the fact that nonhypoxemic patients show less cognitive deterioration [41]. In other words, oxygen-dependent patients have been found to achieve lower scores than controls or nonoxygen dependent COPD patients in cognitive tests [29, 45] which assess verbal memory, delayed recall, and attention [41]. It has also been documented [47] that severely hypoxic patients obtained lower scores than mildly hypoxic patients in simple attention and delayed logical memory tests.

Another important factor that contributes to cognitive decline is hypercapnia or hypercapnea-induced hypoventilation [43]. It has been shown [27] that hypercarbia had a high correlation with information-memory-concentration tests. There are studies that have found a significant correlation between high levels of PaCO_2_ and deficits in reaction time or logical thinking [35], in immediate and delayed memory, in complex attention, in information speed processing, in animal-naming of verbal fluency test [47] and in concentration and orientation [27]. Interestingly, it has been shown [47] that neuropsychological test scores were generally more highly correlated with PaCO_2_ than with PaO_2_.

The role of hypoxia and hypercapnia on cognitive decline can also be proved by the fact that therapy, namely, long-term oxygen therapy (LTOT) or lung volume reduction Surgery (LVRS), seems to improve some cognitive functions [101, 102] such as attention and verbal memory [31, 38] or at least partially reverses them [97]. It has also been suggested that the regular use of oxygen has a neuroprotective role in COPD [96]. However, there are studies that have failed to show any association between gas blood and impaired cognitive functions such as attention [34, 39], language abilities, executive functions [47], mental speed, and fluid intelligence [46, 103].

**Figure 1:**
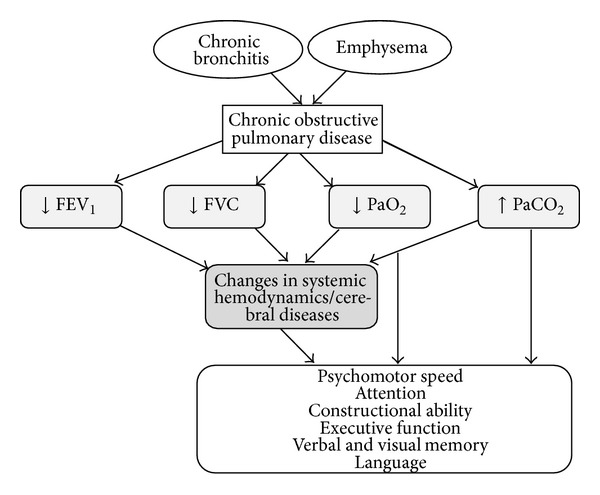
Potential COPD mechanism by which cognitive functions are affected.

The exact pathophysiological and biological mechanisms that could explain the effects of impaired lungs on the development of cognitive function impairments are still unclear. It has been suggested that low lung functioning may contribute to cognitive disorders by decreasing the oxygen delivery to brain neurons. In other words, low arterial oxygen blood pressure and hypercapnia may develop in these patients as a consequence of their disease and as a result, they contribute significantly to the development of pathophysiology in COPD [97]. It has been suggested that impaired lung functioning causes changes in the central nervous system through processes such as cerebral diseases resulting from impaired fibrinolytic activity, oxidate stress, [99] vascular diseases, and increased proinflammatory cytokines such as TNFR1 that act as a possible systemic mediator via cardiovascular disease between lung functions and brain [45]. Increases in the circulating levels of thrombotic factors may be expected to elevate the risk for cardiovascular disease [104]. In other words, cognitive deficits are mediated by the presence of changes in systemic hemodynamic and cerebral diseases. Moreover, it has been suggested that COPD may accelerate aging processes resulting in decreased cerebral blood flow and oxygen consumption [49]. The potential COPD mechanism by which cognitive functions are affected is summarized in Figure 1.

### 4.2. Mechanism of Cognitive Deficits in OSAS Patients

The cause of cognitive deficits in OSAS patients is more complicated than in COPD patients. Some researchers have shown a significant correlation between cognitive impairment and daytime sleepiness related to sleep defragmentation resulting from frequent apneas [105, 106], while others attribute cognitive decline to nocturnal hypoxemia [85, 88]. Finally, some researchers attribute cognitive impairments to a combination of PSG parameters, namely, AHI, sleep arousals, oxygen desaturation, and sleep architecture [7, 107].

It has been suggested that cognitive deficits, such as memory impairment and attentional deficits are affected by sleep defragmentation, but impairments in executive functions, in constructional abilities, in motor tasks and in language are caused by hypoxemia [57, 108, 109]. More specifically, several studies found that vigilance impairment derives from changes in sleep architecture [77, 110] that in turn contribute to memory decline [110, 111]. It has also found [88] that sleep architecture (percentage of SWS sleep and REM) is associated negatively with immediate memory and low level memory scanning. In addition, a study [84] showed that the best predictor of episodic memory deficit was the number of microarousals.

Regarding the effects of hypoxemia on neurocognitive functions, it has been found to be related to psychomotor speed impairments [98, 112]. It was observed [7] that a more severe oxygen desaturation is associated with poorer motor performance and lower processing speed as well as executive dysfunctions [110]. Constructional disabilities have been found to associate with oxygen saturation levels below 80% [85]. A significant correlation was found [60] between the time of oxygen saturation levels below 90% or the lowest peaks of SaO_2_ and deficits in phonemic fluency in severe OSAS patients. It has been reported that working memory and high-speed memory scanning deficits, factors pertaining to executive functions, are associated with the degree of hypoxemia (mean SaO_2_) [85, 88].

However, the mechanism of cognitive deficits in OSAS is still unclear and the results of the researches are complicated and equivocal. For example, there are studies that link hypoxemia with attention [112] and delayed recall [85] and on the other hand, sleep architecture is related to language and executive functioning [87]. Other researches failed to associate executive functions, such as working memory, with hypoxemia [83]. It has also been suggested that specific cognitive deficits, such as verbal delayed memory, are associated with both sleep defragmentation and oxygen desaturation [59, 85]. Some studies do not ascribe cognitive dysfunctions to both hypoxemia and sleep deprivation, especially in tasks that assess verbal short-term and long-term memory, nonverbal memory, constructional abilities, language [8, 79, 89], attention, and executive functions [8, 78, 93].

**Figure 2:**
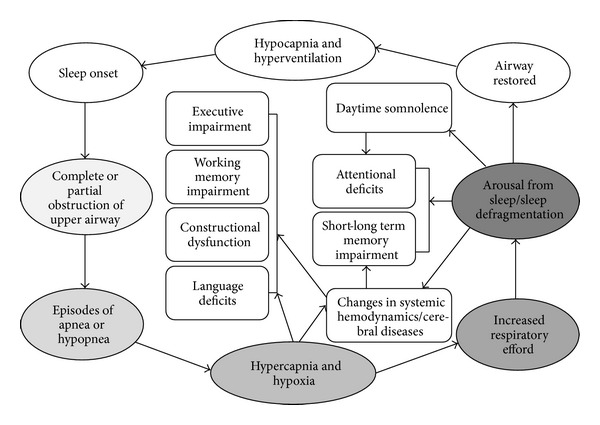
The possible minimal mechanism that causes cognitive deficits in OSAS patients.

As previously mentioned, OSAS patients are presented with significant nocturnal hypoxia, changes in sleep architecture as well as with hypercapnia, and cortical and sympathetic activation [113, 114]. These events cause cardiovascular, cerebrovascular, and metabolic disease as well as sudden death. More specifically, repetitive episodes of deoxygenation and reoxygenation increase the production of proinflammatory cytokines (TNF), C-reactive protein, and chemoreceptors substances that have been associated with the development of atherosclerosis and high arterial pressure [113, 114]. Finally, OSAS patients present blood flow reduction during apneic episodes in left frontal and temporal lobes [59], in prefrontal and parietal cortex, and in middle cingulated area [58]. In conclusion, the exact biological mechanism that could explain the effects of OSAS on cognitive functions should include hypoxemia, sleep deprivation, and changes in cerebral hemodynamic as well as cerebral diseases. The potential OSAS mechanism by which cognitive functions are affected is summarized in Figure 2.

### 4.3. The Role of Hypoxemia, Hypercapnia and Sleep Defragmentation on Cerebral Cognitive Functions

As previously shown, COPD and OSAS patients are presented with common neurocognitive deficits. It has also been noted that hypoxemia is present in both these respiratory disorders and it is associated with the majority of cognitive impairments. So, we may claim that the cognitive impairments in both diseases are mainly based on hypoxemia and secondary to hypercapnia, changes in systemic hemodynamic, cerebral diseases, and sleep deprivation.

Regarding hypoxemia, it has been found that decreased oxygen transport to brain cells modifies ion channels (potassium, sodium, calcium) and increases glomus cell excitability. In addition, it causes alterations in neurotransmitters (dopamine, acetylcholine, and ATP) and reduces respiration followed by enhancement of sympathetic and respiratory activities which results in changes involving modification in signaling pathways, in neuromodulators (endothelin-1) and their receptors and genome effects [6, 115]. Therefore, brain oxygenation is essential for neural biosynthetic processes situation [6]. It has been found that frequent oxygen desaturation during everyday activity elevates choline level in frontal brain areas that damage myelin and membrane precursors [45]. It is remarkable that even low normal range blood oxygen levels may cause cognitive decline [6]. Moreover, oxygen desaturation may increase hematocrit levels and cause angiogenesis through enhancement of production of vascular growth factors [6]. Finally, it has been claimed that hypoxemia, inflammation, and oxidate stress initiate the process of endothelian dysfunction that plays a significantly role in vascular tone and cellular growth [116]. In relation to the negative impact of hypoxemia on brain regions, it has been found that there is a significant decreased cerebral perfusion in left middle and superior frontal, right superior frontal and left parietal lobes [41], and in subcortical regions [43] that are related to attention, verbal memory, executive functions, and psychomotor speed [117]. Some regions of the brain, such as the hippocampus, basal ganglia, cerebellum, occipital cortex, and some frontal regions are particularly susceptible to oxygen deprivation than others [118]. Studies on hypobaric hypoxemia also found a neuronal loss in hippocampus [119]. Furthermore, magnetic resonance image studies revealed a significant gray matter loss and atrophy in several brain regions including cortex, hippocampus, and striatum suggesting that chronic hypoxemia results in neuronal damage and therefore impaired cognitive functions [120, 121].

Regarding changes in sleep architecture, it is well established that chronic sleep deprivation or sleep fragmentation in healthy subjects may cause a similar decline in alertness, simple attention, psychomotor speed as well as learning, memory, working memory, and executive functions [122–124]. Functional magnetic resonance imaging (FMRI) has shown the negative impact of sleep deprivation on specific brain regions, such as temporal to parietal regions and frontal lobe regions. In addition, it has been suggested that long-term sleep deprivation may cause permanent neuronal alterations especially in memory related brain regions, such as the hippocampus [125] as well as reduced activation of frontal and parietal networks and altered functioning within the thalamus [122]. Moreover, it has been found that both short-term and long-term sleep deprivation are associated with the upregulation of hundred of genes in the cerebral cortex and other brain areas. They include genes that are associated with energy metabolism, memory formation, memory consolidation, protein synthesis, and synaptic depression [126].

As previously seen, hypoxemia and sleep defragmentation cause cellular and molecular changes that lead to disruption of functional homeostasis and altered neuronal and glial viability within particular brain regions [57, 127], such as frontal lobe [94, 128, 129] and temporal cortex [58]. Moreover, hypoxemia and sleep deprivation modulate the expression of inflammatory mediators such as interleukins and tumor necrosis factor alpha [116].

OSAS and COPD patients may also suffer from hypercapnia. High carbon blood levels increase cerebral blood flow (CBF) by 25 to 40% [130], while at the same time a profound cortical desynchronization occurs that lasts for the duration of the hypercapnic episode and returns to baseline soon after cessation [131].

However, hypoxia, hypercapnia, sleep defragmentation, or smoking, hemoglobin levels, inflammation parameters, and comorbidities are unlikely to account for all the cognitive dysfunctions [132]. Decline in language abilities and in verbal memory may be associated with age and duration of hypoxemic-hypercapnic chronic respiratory failure [43]. It was found [8] that younger OSAS patients are more sensitive than older OSAS patients to sleep defragmentation and hypoxemia. Another study found that socioeconomic status and adolescent cognitive ability may explain verbal disability, verbal memory decline, or the rate of impairment in memory [99]. In addition, the positive effect of improved quality of life in cognitive dysfunctions was demonstrated [37]. Finally, it has been claimed [133] that intelligence and cognitive functions are interrelated. In other words, high-intelligence may have a protective effect against cognitive decline in OSAS patients, due to increased cognitive reserve. Similarly, a lower IQ correlated significantly with lower scores in neurocognitive tests in COPD patients [46].

## 5. Conclusions

Upon a close review of the literature, it is apparent that since most studies are not homogeneous in COPD and OSAS severity, it is difficult to draw safe conclusions about the reasons of the presence of cognitive impairments in attention, memory, psychomotor speed, and executive functions in those two syndromes. Moreover, the diversity of results is due to differences in methodology and in the types of neuropsychological tests which were used to assess cognitive functions. Furthermore, the treatment duration, low tolerance of treatment, and disease duration are not often taken into account. Few are also known about the etiology of COPD and OSAS, while it is difficult to find out to what extent sleep architecture, blood cerebral flow, gas blood, and comorbidities contribute to cognitive impairment.

In this review, we showed that the effects of sleep fragmentation, hypoxemia, hypercapnia, CBF, and vascocerebral diseases on cognitive functions are intermingled and synergistic. Because of the presence of common cognitive deficits and the significant correlation that these impairments have with low blood oxygen levels in both syndromes, it seems that low blood oxygen pressure is the dominant factor that contributes to common cognitive impairments in both COPD and OSAS.

However, further research is needed which may provide evidence to our claim based on the existing literature and explain the presence of common cognitive impairments in both COPD and OSAS patients.

## Figures and Tables

**Table 1:** Studies examining the effects of COPD on cognitive functions.

Study	Sample characteristics	Type of study	Results
Crews et al. 2001 [9]	47 patients, (FEV_1_) < 25% age = 55.45 ± 6.67	Case control—normative study	50% of the patients exhibited impaired memory performance. Clinically notable frequencies of impairment (greater than 20%) were found on language abilities, executive functions, and psychomotor speed.

Antonelli-Incalzi et al. 2007 [28]	149 patients, FEV_1_ = 36.6 ± 17.8%, age = 69.3 ± 8.5	Clinical case—normative study	Patients exhibited a general cognitive decline.

Hung et al. 2009 [29]	4150 patients, 29% severe (oxygen depended or disease related activity limited), age = 62.6 ± 1.8; 71% nonsevere, age = 62.9 ± 1.8	Community sampled case—control study	Only severe COPD was associated with lower cognitive performance

Etnier and Berry 2001 [30]	98 patients, age = 58–80, FEV_1_ = 58.34 ± 16.41%	Exercise intervention follow-up study	Age, aerobic fitness, or pulmonary function are predictive of cognitive performance on fluid intelligence, reaction time, and working memory span.

Borak et al. 1996 [31]	90 patients (severe COPD treated with long-term oxygen therapy)	Clinical case study	Patients presented poor visual, verbal, and spatial memory.

Kozora et al. 2002 [32]	30 patients, age = 66.5, FEV_1_ = 39.9 ± 1.6%	Clinical case—control study	Significant impairment in visual attention, verbal memory, constructional abilities, psychomotor speed, and executive functions.

Kozora and Make 2000 [33]	30 patients (severe COPD, followed 3 weeks of rehabilitation), 29 untreated COPD patients, 21 healthy controls	Clinical case—control study	Significant group differences were found on sustained visual attention, visual memory, and language abilities.

Vos et al. 1995 [34]	39 patients, age = 65.9 ± 5, FEV_1_ = 33 ± 10%, 38 healthy controls	Case—control study	Lower attention performance of COPD patients in comparison to healthy controls.

Klein et al. 2010 [35]	60 patients, age = 63.2 ± 9.8 FEV_1_ = 36.4 ± 12.5%, 60 healthy controls	Clinical case—control study	Significant group differences found in phasic alertness and orienting but not in executive attention. Reaction time was significantly slower in the COPD group. Differences were found in verbal, visual learning, and logical thinking.

Emery et al. 2001 [36]	29 patients, age = 67.8 ± 7.4, FEV_1_ = 43 ± 17%, 29 controls	Clinical study of exercise effects	Only the improved performance in verbal fluency test was associated with exercise.

Kozora et al. 2005 [37]	39 patients, age = 64.8 ± 4.9 FEV_1_% = 24.9 ± 11, 39 healthy controls	Clinical case—control study	Patients with moderate-to-severe emphysema had impaired attention, verbal memory, and constructional abilities.

Watanabe et al. 2001 [38]	1 patient, 71 yrs old, FEV_1_ = 39%	Clinical case study of lung volume reduction surgery	All cognitive functions improved after lung volume reduction surgery.

Orth et al. 2006 [39]	32 patients, age = 57,4 ± 8,2 FEV_1_ = 50,4 ± 18,2%, 10 healthy controls	Clinical case—control study	Patients demonstrated significantly worse results than healthy controls in intelligence, simple, selective, and sustained attention tasks.

Antonelli-Incalzi et al. 2003 [40]	15 patients (COPD with severe hypoxemia), 18 (COPD without hypoxemia), 10 healthy controls	Clinical case—control study	Patients performed below normal in verbal attainment, attention, and deductive thinking tasks.

Ortapamuk and Naldoken 2006 [41]	8 patients (COPD with hypoxemia and hypercapnia), age = 52.6 ± 5.4, 10 patients (with stable COPD), age = 54.8 ± 6.9, FEV_1_ = 33.9 ± 13%, 10 healthy controls	Clinical case—control study	Scores in verbal memory, delayed recall, and attention tasks were significantly lower in COPD patients than healthy controls. No differences in psychomotor speed.

Kozora et al. 1996 [42]	32 patients (COPD with mean partial arterial oxygen pressure 68.8 mm Hg, 73% receiving supplementary oxygen), age = 70.3, 31 healthy controls	Clinical case—control study	COPD patients performed significantly worse than controls in verbal fluency and attention tasks.

Incalzi et al. 1993 [43]	36 patients (severe COPD, receiving oxygen therapy), age = 69 ± 10, 29 normal adults (69 ± 7 yr), 20 normal elderly adults (78 ± 2 yr), 26 patients (Alzheimer-type dementia, (72 ± 2 yr), 28 patients (multi-infarct dementia, 70 ± 8 yr)	Clinical case—control study	48.5% of patients with COPD had a specific pattern of cognitive deterioration characterized by impairment in verbal and verbal memory tasks, well-preserved visual attention, and diffuse worsening of the other functions.

Antonelli-Incalzi et al. 2006 [44]	149 patients, age = 68.7 ± 8.5, FEV_1_ = 36.5 ± 18.0%	Clinical case study	The prevalence of cognitive impairment was 32.8%. Visual memory was not impaired.

Borson et al. 2008 [45]	18 patients (mild to severe COPD), 2 patients (FEV_1_ = 50–79%), 8 patients (FEV_1_ = 30–49%), 8 patients (FEV_1_ < 30%), age = 68.5 ± 8.0, 9 healthy controls	Clinical case—control study	Performance in verbal memory, land psychomotor speed was impaired.

Liesker et al. 2004 [46]	30 patients, FEV_1_ = 49 ± 8% age = 64.8, 20 controls	Clinical case—control study	COPD patients performed significantly worse than controls in psychomotor speed tasks.

Stuss et al. 1997 [47]	18 patients FEV_1_ = 74 ± 30%, age = 66.4 ± 7.6	Clinical case—control study	Intelligence scores and psychomotor speed and memory abilities were within average range.

Antonelli-Incalzi et al. 2009 [48]	54 patients (hypoxemic COPD), age = 68.8 ± 9.0, FEV_1_ = 36.9 ± 16.5%	Clinical study	Patients had impaired performance in copying drawings with landmarks

Incalzi 1997 [49]	42 patients (COPD with hypoxemia and hypercarbia), FEV_1_ = 34 ± 9%, age = 70 ± 9.7, 27 normal subjects, 31 patients (Alzheimer's disease), 26 older normal subjects	Clinical case—control study	The overall cognitive performance of COPD patients was significantly inferior to that of the rest of the groups. Both passive recognition and active recall of learned material were severely impaired in COPD patients.

Fioravanti et al. 1995 [50]	50 patients	Case study	30% of COPD patients had impaired immediate verbal memory.

Kozora et al. 1995 [51]	40 patients, age = 64, 40 healthy controls	Clinical case—control study	COPD patients were significantly impaired during copying.

Kozora et al. 1998 [52]	59 patients, age = 66.7, 31 healthy controls, age = 65.9	Clinical case—control study	COPD patients performed significantly worse than controls in two tasks (verbal fluency and digit span) but not at a clinically impaired range.

**Table 2:** Studies examining the effects of OSAS on cognitive functions.

Study	Sample characteristics	Type of study	Results
Quan et al. 2006 [7]	67 patients, age = 59.4 ± 9.2, AHI = 22.4, AHI > 10, 74 healthy controls	Case—control study	Mild to moderate OSAS has little impact on the selected measures of attention, executive function, and motor and processing speed.

Mathieu et al. 2008 [8]	28 patients, two age groups, Group A: <50 yr, AHI = 50.8 ± 4.1, Group B: >50 yr, AHI = 42.5 ± 4.1, 30 healthy controls	Case—control study	OSAS patients exhibit attention and verbal long-term memory dysfunctions. Short-term memory, working memory, and planning and flexibility appeared well preserved. OSAS patients showed some difficulty in the initial acquisition of the procedural skill but no procedural skill-learning deficit over time.

Lim et al. 2007 [53]	46 patients; 14 patients (OSAS treated with placebo), age = 48.9 ± 3.2, AHI = 65.8 ± 8.2; 17 patients (OSAS treated with CPAP), age = 46.7 ± 2.4, AHI = 63.5 ± 7.8; 15 patients (OSAS treated with oxygen), age = 47.1 ± 2.3, AHI = 58.6 ± 8.3	Randomized placebo—controlled design	OSAS patients showed diffuse impairments, particularly in terms of speed of information processing, attention and working memory, executive functioning, learning and memory, and alertness and sustained attention.

Yaouhi et al. 2009 [58]	16 patients, age = 54.75 ± 5.71, AHI = 38.31 ± 14.33, 14 healthy controls	Case—control study	Patients showed poor performance on episodic memory. The remaining neuropsychological test scores (attention, vigilance, working memory, executive functions, and verbal fluency) were within the normal range.

Ferini-Strambi et al. 2003 [60]	23 patients, age = 56.5 ± 6.13, AHI = 54.9 ± 13.37, 23 healthy controls.	Case—control study	OSAS patients had a significant impairment, compared to controls, in tests of sustained attention, visuospatial learning, executive function, motor performance, and constructional abilities.

Muñoz et al. 2000 [64]	80 patients, age = 49.1, AHI = 60.2, 80 healthy controls	Clinical study of CPAP effects	Patients had a longer reaction time and poorer vigilance than healthy controls.

Engleman et al. 1994 [67]	32 adult patients, AHI = 28 (range 7–129)	Placebo—controlled, crossover study	Patients showed improved vigilance, mental flexibility, and attention after CPAP treatment.

Engleman et al. 1999 [68]	34 patients, age = 44,8 ± 6, AHI = 10 ± 3 (range 5–15)	Placebo control study of CPAP effects	Differences between before CPAP and after CPAP treatment in OSAS patients in attention (improvement in tests such as digit symbol examining attention skills).

Naegele et al. 1998 [69]	17 patients, age = 44.80 ± 2.5, AHI = 53.5 7.6, 17 healthy controls	Case—control study of CPAP effects	Executive and learning disabilities as well as short-term memory impairment were found in OSAS patients.

Andreou and Agapitou 2007 [73]	20 adolescents, age = 18.41 ± 0.37, AHI = 15.48 ± 7.08, 20 healthy controls	Clinical case—control study	Snoring adolescents showed reduced language and verbal abilities which were associated with disruption of sleep by apneas.

Aloia et al. 2004 [74]	37 peer-reviewed articles were selected for this review	Review	Findings were equivocal for most cognitive domains. Treatment was noted to improve attention/vigilance in most studies but not constructional abilities or psychomotor functioning.

Rouleau et al. 2002 [75]	28 patients, 18 healthy controls	Clinical study	OSAS patients did not show procedural skill learning deficits and episodic memory impairments. Frontal dysfunction, decrement in psychomotor efficiency, and vigilance characterized the impaired neuropsychological profile of OSAS patients.

Lau et al. 2010 [76]	37 adult patients (moderate to severe OSAS treated with CPAP) 27 healthy controls	Clinical case—control study	Treated individuals performed at a comparable level to controls on basic working memory storage tests but showed a significant reduction on tests of working memory, requiring central executive functioning. The patients also performed worse on complex attention, executive function, and psychomotor speed tests than healthy controls.

Kingshott et al. 2000 [77]	62 patients, age = 51 ± 11, AHI 62 ± 33	Clinical case study of CPAP treatment	Cognitive performance tests measuring coding speed, reaction time, and attention were found improved after CPAP therapy.

Mazza et al. 2005 [78]	20 patients, age = 51 ± 2 yrs, AHI = 45 ± 22, 40 controls	Clinical case—control study	95% of patients had vigilance and/or attention impairment.

Pierobon et al. 2008 [79]	157 patients, age = 47.8 ± 11.9, AHI = 54.4 ± 0.9	Clinical case—normative study	Patients were impaired in short-term verbal memory and in short-term visual spatial memory. 40.8% did not have cognitive deficits.

Monasterio et al. 2001 [80]	142 patients, age = 53 ± 9 AHI = 10–30	Clinical case—control study	All the initial mean values of the cognitive tests performed were not impaired.

Twigg et al. 2010 [81]	60 patients, age = 51 ± 9, AHI = 23.1, 60 healthy controls.	Clinical case—control study	Patients with OSAS displayed reduced performance on verbal episodic memory tasks, whereas visual episodic, semantic, and working memory remained intact.

Dècary et al. 2000 [82]	Review	Review	Poor general intellectual functioning, attention, memory and learning abilities, executive functions, and motor performance were found in OSAS patients.

Felver-Gant et al. 2007 [83]	56 patients, age = 52.8 ± 11.2, AHI = 41.4 ± 22.1	Clinical case—control study	Working memory, declarative memory, executive functioning, and motor speed was impaired in OSAS patients.

Daurat et al. 2008 [84]	28 patients, 29 healthy controls	Clinical case—control study	Recollection was strongly disturbed in patients, while attention was only slightly disturbed.

Aloia et al. 2003 [85]	12 patients, age = 64.8 ± 6.4, AHI = 51.2 ± 19.8	Clinical case study	Attention, verbal delayed recall, constructional abilities, and language were impaired in OSAS patients.

Feuerstein et al. 1997 [86]	10 patients, 10 healthy controls	Clinical case—control study of CPAP treatment	Patients were found with a significant decreased ability to initiate new mental processes and to inhibit automatic ones in conjunction with a tendency for perseverative errors. Deficits of verbal and visual learning abilities.

Lee et al. 2009 [87]	30 patients, 30 healthy controls	Clinical case—control study	More severe cases of OSAS were associated with impaired language function reflecting frontal-subcortical pathology.

Grenèche et al. 2011 [88]	12 patients, age = 51.8 ± 2.5, AHI = 58.9 ± 11.4, 10 healthy controls	Clinical case—control study	OSAS patients exhibited poor working memory performances. Immediate memory was not impaired.

Torelli et al. 2011 [89]	16 patients, age = 55.8 ± 6.7, AHI = 52.5 ± 26.0, 14 healthy controls	Clinical case—control study	Patients with OSAS had impaired auditory-verbal learning. No other significant differences between the two groups were detected.

Saunamäki et al. 2009 [90]	40 patients, age = 47.2 ± 7.8, AHI = 41.0 ± 22.8; 20 healthy controls.	Clinical case—control study	Patients showed poorer performance than controls on executive functions.

Bardwell et al. 2001 [91]	36 patients, Group A (placebo group), age = 48 ± 2.2, AHI = 43.6 ± 6.4, Group B (CPAP), age = 47 ± 1.9, AHI = 56.8 ± 5.4	Clinical study of CPAP effects	OSAS patients exhibited poor executive functions.

Salorio et al. 2002 [92]	28 patients, age: 28–60 yrs, 24 healthy controls	Clinical case—control study	Patients exhibited poorer recall abilities across learning trials, less efficient use of semantic clustering, and poorer use of semantic cues. Retention of previously encoded information and recognition was intact. No deficits in general executive control.

Lis et al. 2008 [93]	20 patients, age = 53.4 ± 10.5, AHI = 57.9 ± 20.2; 10 healthy controls	Clinical case—control study	Deficits in tasks of executive functioning.

Beebe et al. 2003 [94]	25 studies. 1092 patients, 899 healthy controls.	Meta-analytic review of research through 2001	OSAS was found to have a negligible impact on intellectual and verbal functioning but a substantial impact upon vigilance and executive functioning. Data were mixed with regard to visual and motor functioning. Post-hoc inspection of the data suggested that tests of fine-motor coordination or drawing were more sensitive to OSAS than were tests of fine-motor speed or visual perception. Data were also mixed with regard to memory functioning.
